# A microsatellite polymorphism in IGF1 gene promoter and longevity in a Han Chinese population

**DOI:** 10.1186/1756-0500-3-55

**Published:** 2010-03-03

**Authors:** Liang Xie, Yuan-ying Gong, Shi-gang Lian, Juan Yang, Shou-jun Gao, Liang-you Xu, Ya-ping Zhang

**Affiliations:** 1 State Key Laboratory of Genetic Resources and Evolution, Kunming Institute of Zoology, Chinese Academy of Sciences, Kunming 650223, China; 2 Laboratory for Conservation and Utilization of Bio-resource, Yunnan University, Kunming 650091, China; 3 Graduate School of the Chinese Academy of Sciences, Beijing 100049, China; 4 People's Hospital of Dujiangyan City, Dujiangyan 611830, China; 5 Dujiangyan Longevity Research Centre, Dujiangyan 611830, China

## Abstract

**Background:**

Previous studies have suggested a probable association between the polymorphism of a microsatellite locus located in the promoter of IGF1 (Insulin-like growth factor 1) gene and the serum level of IGF1, as well as many age-related diseases. Based on these results, we hypothesized that this polymorphism may influence longevity in humans. We performed an association study in a Han Chinese population to test this hypothesis.

**Findings:**

We recruited 493 elderly Han Chinese individuals (females ≥ 94; males ≥ 90) and 425 young individuals (controls) from Dujiangyan (Sichuan province, China). The genotype distributions and allele frequencies of the microsatellite site in the elderly and control groups were compared by chi square test.

Our results suggested that there was no association between the microsatellite polymorphism and longevity in our Han Chinese population. However, there were more male persons with 18/21 genotype in elderly group than that in control group (11.11 vs. 5.45%, *p *= 0.011). As the difference was not significant when corrected by Bonferroni method, we speculate that the 18/21 genotype can not be functional in longevity; however, it may link with the real functional loci as there is a long haplotype block embracing the microsatellite locus.

**Conclusions:**

There was no association between polymorphism of the microsatellite in promoter of IGF1 gene and longevity in our study. Future association studies containing the long haplotype block are deserved and can test our speculation of the potential linkage of 18/21 genotype and functional loci.

## Background

IGF1 (Insulin-like growth factor 1) plays a number of important roles in the human body. It is involved in physiological processes such as growth, development, and metabolism [1], and has been implicated as a factor in the development of common diseases [2]. Meanwhile, a conserved insulin/IGF1 signal pathway which may affect ageing and longevity is known in *E.coli*, yeast, drosophila, mice and humans [3-7]. Elucidating the functions of insulin/IGF1 pathway in ageing and longevity is a current hot spot in longevity research [8].

Serum IGF1 levels appear to influence susceptibility to disease, and therefore longevity. For example, people with a high level of circulating IGF1 are more susceptible to cancers, while a low level of circulating IGF1 is a risk factor for cardiovascular diseases, premature atherogenesis, and diabetes [5]. Furthermore, Yang et al. suggested that in humans, a reduced level of serum IGF1 in early adulthood and an increased level in elderly time might be a predictor of high longevity. Meanwhile, the heritability of individual IGF1 level variation is about 50% [9], which implies that IGF1 regulation is genetically determined. Considering the evidence for heritability of longevity [10], perhaps there is a hereditable shift in IGF1 regulation in long-lived people [8].

A cytosine-adenine (CA) repeat polymorphism in IGF1 gene promoter region (1 kilo base pairs upstream from the transcription start site) [11] has been associated with serum IGF1 level [12,13]. At the same time, many studies have related this microsatellite polymorphism to age-related diseases such as diabetes, cancers, and cardiovascular diseases [14-19]. These results are disputed by several other studies [9,20-24]. However, they suggest that this microsatellite locus might be a potential functional site for serum IGF1 level regulation, and might therefore function in ageing and longevity. Therefore, exploring the association of this microsatellite polymorphism and longevity is urgent.

To our knowledge there have been no reports on the association between the microsatellite polymorphism and longevity in the Han Chinese people. In this study, we conducted an association study in a Han Chinese population to investigate the potential function of this microsatellite locus in predicting longevity.

## Methods

### Subjects

A total of 493 elderly individuals (females ≥ 94 years old; males ≥ 90 years old) from Dujiangyan (Sichuan province, China) agreed to participate in this study. Ages were authenticated by the official certification of the Fifth Nation Census in China as well as by the inference of the number of generations, and the memories of these, their offspring and other local elderly people. The age was confirmed when the certification and the inference were consistent. We also recruited 425 healthy, local individuals between the ages of 32 - 73 years to act as a control group. This control group included 110 spouses of the children of the elderly participants. The subjects' information is listed in Table 1.

**Table 1:** Demographics of the study participants

Group	N (Male/Female)	Age (Mean ± SD, years)	Nationality	Range of age (year)
Elderly	493 (252/241)	94.97 ± 3.15	Han	Male ≥ 90; Female ≥ 94
Control	425 (202/223)	57.17 ± 9.21	Han	32-73

All participants indicated informed consent by signing a form after they had been given a clear explanation of the potential risks of the study. This research was approved by the Ethics Committee on human experimentation of Kunming Institute of Zoology, Chinese Academy of Sciences and relevant bodies in Dujiangyan. The study was done in accordance with the Declaration of Helsinki and subsequent amendments.

### Genotyping

DNA was extracted from white blood cells in whole blood by the standard phenol/chloroform method. Primers for PCR followed those used in previous study [21]. The forward primer was labeled at the 5'-end with 6-FAM fluorescent dye. PCR reactions each included 20 ng genomic DNA as template, 2.5 μL 10× PCR buffer, 1.0 μL dNTP (2.5 mmol·L^-1^), 0.5 μL BSA (bovine serum albumin, 200 μg·ml^-1^), 0.5 μL forward primer (10 pmol·μL^-1^), 0.5 μL reverse primer (10 pmol·μL^-1^), 0.125 μL rTaq polymerase (Takara, Dalian, China) and distilled water to a total volume of 25 microliter (μL). Thermocycling parameters included an initial denaturation of 5 min. at 95°C; 35 cycles of: 95°C for 30 s, 62°C for 1 min. and 72°C for 1 min.; and a final extension at 72°C for 10 min. PCR products were denatured and size fractionated on 6% denaturing polyacrylamide gel run on an ABI 3730 automated sequencer (AppliedBiosystems, Foster City, CA). Alleles were read and scored by GeneMapper V4.0 software (ABI Perkin Elmer) using Liz500 as size standard. Alleles were verified artificially twice by two different persons. The number of repeats contained in alleles of different length was confirmed by cloning and sequencing two differently sized PCR products (ie. two alleles). We replicated PCR amplication and genotyping of 30% of the samples, ensuring that replicates were performed by a different researcher. Ambiguous results were confirmed by another PCR and fragment analysis.

### Statistical analysis

A test of Hardy Weinberg Equilibrium (HWE) was performed using Genepop 3.4 [25]. The program PHASE v2.1 [26,27] was used to estimate the haplotype of each individual. Statistical analyses were performed with SPSS (version 13.0, Chicago, IL). Allele distributions and genotype frequencies of the elderly (test) and young (control) groups were compared with Pearson's chi square test, using a two-tailed *p *value was and considering *p <*0.05 to be significant. We used a Bonferroni correction to correct for multiple tests of the same data.

To explore the potential function of this microsatellite locus, we categorized the whole population into three genotype groups depending on owning 2, 1, or no copies of major alleles (19- and 21-repeat): 19/19 (21/21), 19/non (21/non), and non/non. We performed chi square test to compare 1) each genotype to the sum of the other two, 2) the frequencies of three genotypes, and 3) the distribution of major allele (19(21)-allele vs. non) in elderly and control groups in order to detect any potential functions of the major alleles.

## Results

In this study, we examined the microsatellite polymorphism in IGF1 gene promoter in 493 elderly individuals (241 females and 252 males) and 425 control individuals (223 females and 202 males). The length range of the microsatellite locus was 17 to 23 repeats (only one person has a 13-repeat allele), and the most common alleles are 19-repeat (39.12%) and 21-repeat (26.14%). The genotype distribution followed Hardy-Weinberg Equilibrium (HWE) in both elderly and control groups, as well as when stratified by gender (*p *> 0.05).

All alleles and their frequencies are listed in Table 2. Allele frequencies of elderly and control groups were not significantly different (*p *= 0.922, df = 9), even when stratified by gender. The results of comparison of the distribution of common alleles (19- and 21-repeat) in both groups are listed in Table 3. There were no significant differences in genotype distribution and allele frequencies in the two groups considered as a whole. However, when we stratified the groups by gender, there were more individuals with genotype 21/non (21-repeat/non-21-repeat) in the male elderly group than in the male control group (42.06 vs. 32.18%, *p *= 0.031). After Bonferroni correction, the result was no longer significant.

**Table 2:** Allele frequencies of the microsatellite in sampled elderly and control groups.

Allele^1^	All		Women		Men	
	Elderly(%)	Control(%)	Elderly(%)	Control(%)	Elderly(%)	Control(%)
13	1(0.10)	0	1(0.21)	0	0	0
16	1(0.10)	1(0.12)	0	1(0.22)	1(0.20)	0
17	79(8.01)	68(8.00)	37(7.68)	34(7.62)	42(8.33)	34(8.42)
18	169(17.14)	138(16.24)	80(16.60)	71(15.92)	89(17.66)	67(16.58)
19	380(38.54)	338(39.76)	198(41.08)	173(38.79)	182(36.11)	165(40.84)
20	69(7.00)	54(6.35)	29(6.02)	28(6.28)	40(7.94)	26(6.44)
21	260(26.37)	220(25.88)	124(25.73)	121(27.13)	136(26.98)	99(24.51)
22	23(2.33)	28(3.29)	12(2.49)	15(3.36)	11(2.18)	13(3.22)
23	4(0.41)	2(0.24)	1(0.21)	2(0.45)	3(0.60)	0
24	0	1(0.12)	0	1(0.22)	0	0
	*p *= 0.922		*p *= 0.948		*p *= 0.455	

1. The number for an allele is the amount of repeats of this allele.

**Table 3:** Chi square test results for the genotype distribution and allele frequencies of the 19- and 21-repeat alleles in elderly and control groups.

	All			Women			Men		
	Elderly(%)	Control(%)	P value	Elderly(%)	Control(%)	P value	Elderly(%)	Control(%)	P value
Genotype									
19-repeat^1^			0.543			0.582			0.283
19/19^2^	69(14.00)	70(16.47)	0.297	36(14.94)	33(14.80)	0.966	33(13.10)	37(18.32)	0.126
19/non^2^	242(49.09)	198(46.59)	0.45	126(52.28)	107(47.98)	0.355	116(46.03)	91(45.05)	0.835
non/non^2^	182(36.92)	157(36.94)	0.994	79(32.78)	83(37.22)	0.316	103(40.87)	74(36.63)	0.357
Allele									
19-repeat^3^	380(38.54)	338(39.76)	0.592	198(41.08)	173(38.79)	0.477	182(36.11)	165(40.84)	0.145
non	606(61.46)	512(60.24)		284(58.92)	273(61.21)		322(63.89)	239(59.16)	
Genotype									
21-repeat^4^			0.31			0.88			0.082
21/21^2^	30(6.09)	33(7.76)	0.316	15(6.22)	16(7.17)	0.682	15(5.95)	17(8.42)	0.308
21/non^2^	200(40.57)	154(36.24)	0.179	94(39.00)	89(39.91)	0.842	106(42.06)	65(32.18)	**0.031^6^**
non/non^2^	263(53.35)	238(56.00)	0.421	132(54.77)	118(52.91)	0.688	131(51.98)	120(59.41)	0.114
Allele									
21-repeat^5^	260(26.37)	220(25.88)	0.813	124(25.73)	121(27.13)	0.628	136(26.98)	99(24.50)	0.397
Non	726(73.63)	630(74.12)		358(74.27)	325(72.87)		368(73.02)	305(75.50)	

1. P values were obtained by the chi-square test of whole genotype distribution categorized by 19-repeat allele.

2. P values were obtained by the chi-square test of genotype distribution categorized by each genotype.

3. P values were obtained by the chi-square test of allele frequencies categorized by 19-repeat allele.

4. P values were obtained by the chi-square test of whole genotype distribution categorized by 21-repeat allele.

5. P values were obtained by the chi-square test of allele frequencies categorized by 21-repeat allele.

6. After Bonferroni correction, p < 0.0017 is considered significant.

To investigate potential causes of the different frequencies of the genotype 21/non in male participants, we compared the distributions of all possible genotypes in the male elderly and male control groups. Altogether, we found six genotypes which included one allele with 21 repeats: 17/21, 18/21, 19/21, 20/21, 21/22, and 21/23 (The number represents how many repeats there is in the microsatellite locus in this genotype). A chi square test showed a significant difference in the distribution of the 18/21 genotype in male elderly and male control groups (11.11 vs. 5.45%, *p *= 0.011; Table 4). After Bonferonni correction for multiple tests, the result was no longer significant.

**Table 4:** The comparison of genotypes with one 21-repeat allele in male elderly and male control groups

Genotype	Male		
	Elderly(%^1^)	Control(%^1^)	P value^2^
17^3^/21	14(5.56)	11(5.45)	0.716
18/21	28(11.11)	11(5.45)	0.011^4^
19/21	42(16.67)	34(16.83)	0.591
20/21	14(5.56)	7(3.47)	0.182
21/22	7(2.78)	2(0.99)	0.18
21/23	1(0.40)	0	

1. The percentage was obtained from dividing the amounts of people in each category by the total number of people of the respective group including all genotypes.

2. P values were obtained by the chi-square test of genotype distribution categorized by each genotype in respective group.

3. The number represents how many repeats there is in the microsatellite locus in this genotype.

4. The new cutoff p-value is 0.0083.

In a previous study of the same population [28], we reported no association between longevity and the three SNPs (rs2288377, rs5742612, and rs35767) surrounding the microsatellite. Combining the two datasets, we constructed the haplotype of each individual for the microsatellite locus and these three SNPs. The result is listed in Table 5. There are 18 different haplotypes in the population and no significant result was obtained in this study.

**Table 5:** The comparison of haplotype distribution in elderly and control groups.

	All		Female		Male	
Haplotype	Elderly(%)	Control(%)	Elderly(%)	Control(%)	Elderly(%)	Control(%)
C 13^1 ^TT	1(0.10)	0	1(0.21)	0	0	0
C 16 TT	0	1(0.12)	0	1(0.23)	0	0
C 17 TT	78(7.99)	68(8.46)	37(7.71)	34(7.91)	41(8.27)	34(9.09)
C 18 TT	162(16.59)	125(15.55)	74(15.42)	66(15.35)	88(17.74)	59(15.78)
C 19 TT	357(36.58)	295(36.69)	186(38.75)	150(34.88)	171(34.48)	145(38.77)
C 19 CA	1(0.10)	0	0	0	1(0.20)	0
C 20 TT	28(2.87)	21(2.61)	13(2.71)	12(2.79)	15(3.02)	9(2.41)
C 21 TT	10(1.02)	8(1.00)	1(0.21)	3(0.70)	9(1.81)	5(1.34)
T 18 CA	8(0.82)	3(0.37)	6(1.25)	2(0.47)	2(0.40)	1(0.27)
T 19 TT	12(1.23)	8(1.00)	8(1.67)	5(1.16)	4(0.81)	3(0.80)
T 19 CA	5(0.51)	16(1.99)	2(0.42)	11(2.56)	3(0.60)	5(1.34)
T 20 TT	29(2.97)	22(2.74)	12(2.50)	12(2.79)	17(3.43)	10(2.67)
T 20 CA	10(1.02)	7(0.87)	4(0.83)	3(0.70)	6(1.21)	4(1.07)
T 21 TT	6(0.61)	10(1.24)	2(0.42)	8(1.86)	4(0.81)	2(0.53)
T 21 CA	242(24.80)	186(23.13)	121(25.21)	103(23.95)	121(24.40)	83(22.19)
T 22 CA	23(2.36)	28(3.48)	12(2.50)	15(3.49)	11(2.22)	13(3.48)
T 23 CA	4(0.41)	4(0.50)	1(0.21)	4(0.93)	3(0.60)	0
T 24 CA	0	2(0.25)	0	1(0.23)	0	1(0.27)
Total^2^	976	804	480	430	496	374
P-value	0.260		0.142		0.797	

1. The number represents how many repeats there is in the microsatellite locus in this haplotype.

2. The total amounts are less than the whole numbers of the population individuals because there are missing data in SNPs dataset.

## Discussion

In the present study, we explored the association of a microsatellite polymorphism in IGF1 gene promoter with longevity in a Han Chinese population. The elderly and control groups were recruited from the same area. They shared the same living conditions, diet habits, and culture. This allows us to exclude non-genetic factors which may also affect longevity, and ensures that population stratification can be avoided to a certain extent in our study [29]. Additionally, the genotype distributions of both groups did not deviate from HWE.

To our knowledge, this is the first study of the correlation between this microsatellite polymorphism and longevity in a Han Chinese population. The allele distribution of this microsatellite in our population is consistent with a previous study on breast cancer in Chinese people [17]. The distribution is different from that in Caucasian population, as described by Jernstrom et al. [13], demonstrating that allele frequencies at this locus differ between the two populations. This difference may be caused by demographic geography and history of Chinese and Caucasian populations, or by genetic drift. However, within the Han population there is no significant difference in genotypes between elderly and control groups, even when stratified by gender. Our results suggest that no specific alleles at this locus are associated with longevity in this Han population.

To explore the potential role of the microsatellite locus, we compared the genotype frequencies in elderly and control groups depending on 19-repeat and 21-repeat (the two most common alleles) to define categories. As stated in the introduction, the common 19-repeat allele is thought to be related to serum IGF1 levels and many age-related diseases, although the results are equivocal. The contradiction between studies can be explained by different population size, different ethnicities in different studies, and small effect of this polymorphism in complex diseases. Similarly, longevity is a complex trait, influenced by the additive effects of many genetic variations (not to mention stochasticity). Thus an association study may simply not have sufficient power to detect significant correlations between the 19-repeat and longevity.

We initially found a significant difference between the 21-repeat/non genotype frequencies in male elderly (42.06%) and control (32.18%) groups, as well as between the 18/21 genotype (11.11 versus 5.45%, *p *= 0.011) and other genotypes. However, Bonferroni corrections increased the *p *values past the cut-off point for statistical significance. Based on these results, the 21-repeat can not be a potential functional allele for male longevity. The 18/21 genotype may still be linked to real functional variations, because in a recent study, a long haplotype block including this microsatellite region was reported [30]. We constructed haplotypes for each participant using our microsatellite and three SNPs data, and found that no single haplotype was positively associated with longevity. The haplotype block is long enough that more SNPs from this region could be included for testing if a potential functional site exists, and we recommend this approach be used before our initial hypothesis is fully rejected., in order to ensure that small sample size (of loci) did not bias the results.

In summary, the results from the present research suggest no association between the microsatellite polymorphism in IGF1 gene promoter and longevity in a Han Chinese population. The possibility of a functional site linked to the microsatellite locus (especially in men) deserves future studies.

## Conclusions

In this study, no association was discovered between the microsatellite site in IGF1 gene promoter and longevity in a Han Chinese population. In males, the 18/21 genotype was more frequent in the elderly group than in the control group (11.11 vs. 5.45%, *p *= 0.011). Future studies including an increased sample size and more loci in the long haplotype block will enable us to test the hypothesized linkage between the 18/21 genotype and functional loci.

## Competing interests

The authors declare that they have no competing interests.

## Authors' contributions

LX and YPZ designed the research and wrote the manuscript. LX conducted the genotyping and statistical analysis. YG assisted in revising the manuscript. YG, SL, JY and LyX collected the blood samples. All authors read the paper and approved the final version.
